# Supplementary material for: Elucidating the tunability of binding behavior for the MERS-CoV macro domain with NAD metabolites
Source: Commun Biol. 2021 Jan 27;4:123. doi: 10.1038/s42003-020-01633-6 (PMC7840908; doi:10.1038/s42003-020-01633-6)

Supplementary Information for:
Elucidating the tunability of binding behavior for the MERS-CoV macro domain with NAD metabolites

Lin et al.

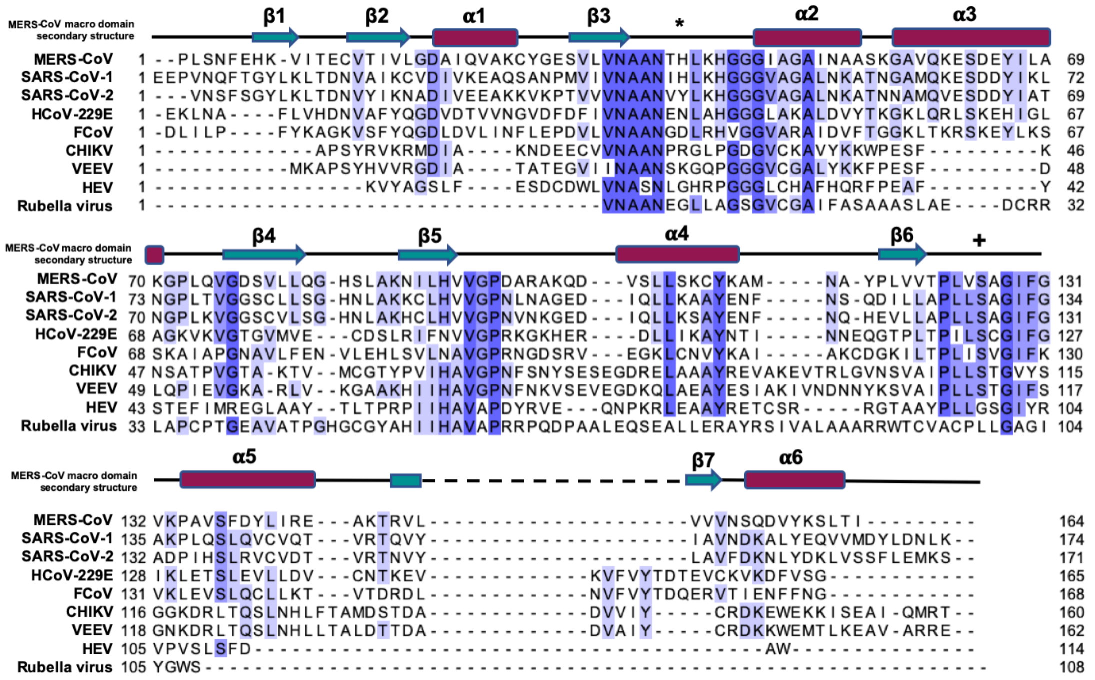

Supplementary Figure S1. Sequence comparison of viral macro domains. Sequence alignment of viral macro domains. The shows are sequences of macro domains from the Middle East respiratory syndrome coronavirus (MERS-CoV), severe acute respiratory syndrome coronavirus 1 and 2 (SARS-CoV 1 and 2), human coronavirus 229E (HCoV-229E), feline coronavirus (FCoV), chikungunya virus (CHIKV), Venezuelan equine encephalitis virus (VEEV), Hepatitis E virus (HEV), and Rubella virus. Secondary structures of the MERS-CoV macro domain are presented at the top of the alignment. Distinguished loops 1 and 2 described in this study are labeled as * and +.

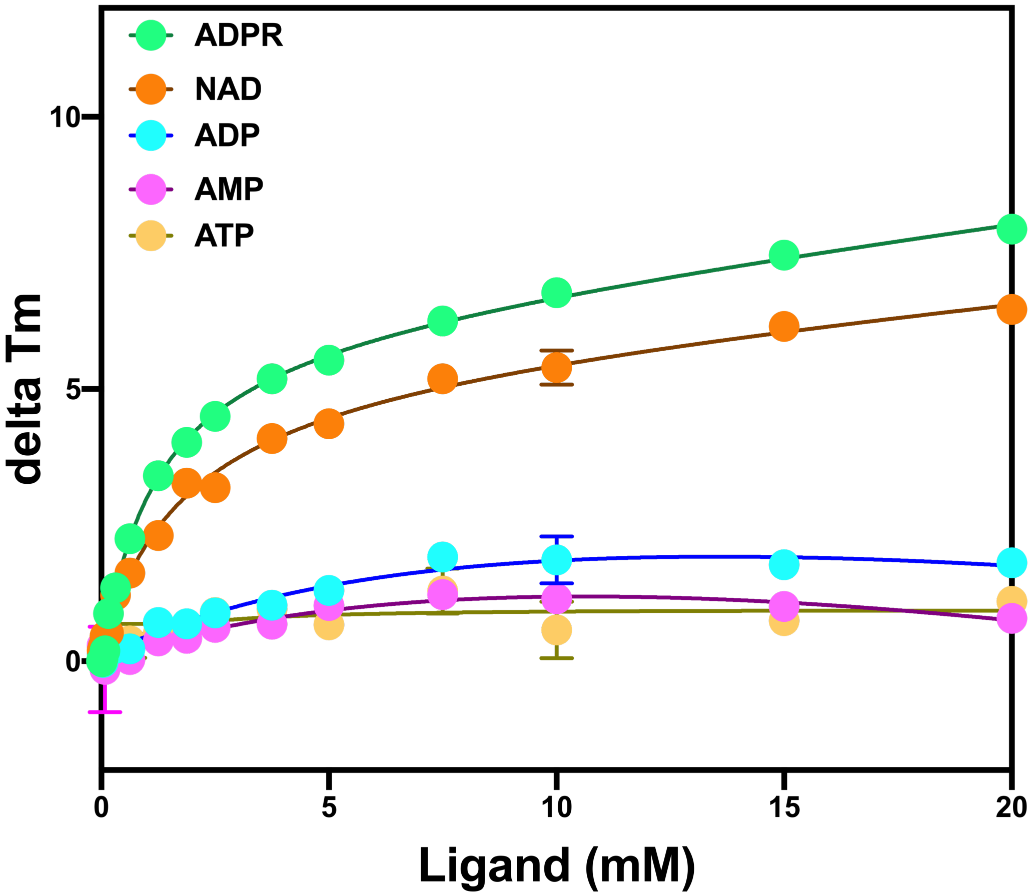

Supplementary Figure S2. NAD metabolites binding to MERS-CoV macro domain, as probed via Differential scanning fluorimetry (DSF). DSF thermal shift assay of 0.2 mM MERS-CoV macro domain incubating with increasing concentrations of NAD metabolites. Data are the mean ± SE of the melting temperature difference (delta Tm) from 3 independent experiments. Data were fitting by the means.

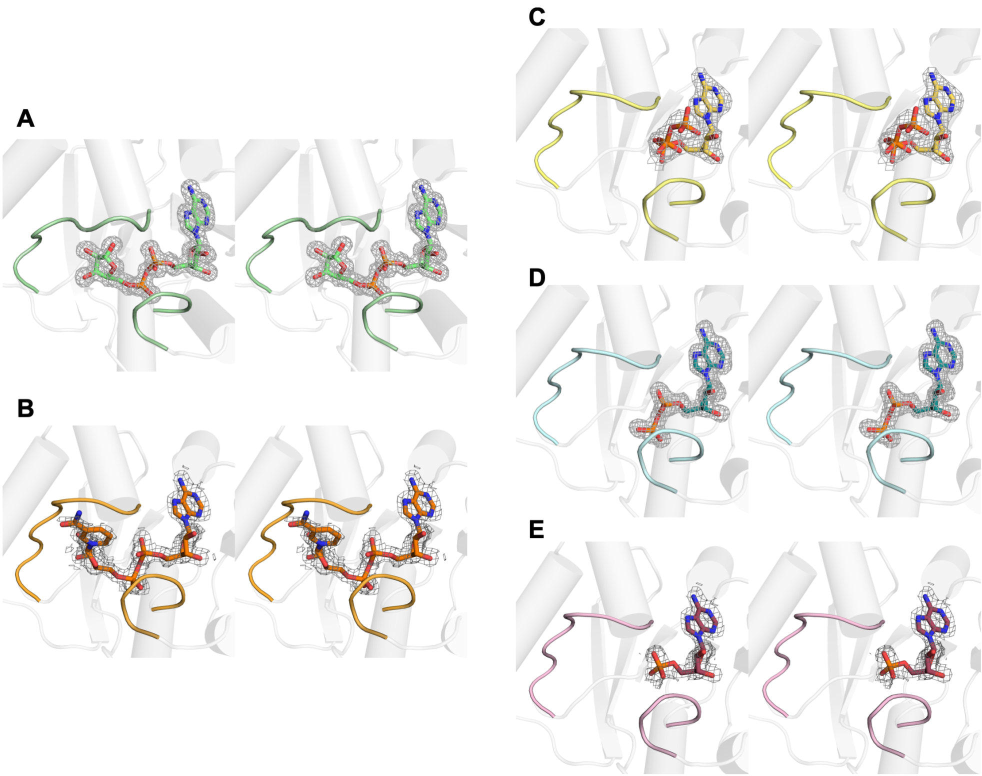

Supplementary Figure S3. Stereo diagrams of NAD metabolites bound in MERS-CoV macro domain ligand binding site with 2Fo-Fc electron density map contoured at 1.0 σ. ADPR (A), NAD (B), ATP (C), ADP (D) and AMP (E) are presented in stick models respectively with carbons in light green, orange, yellow, cyan and light pink. Two divergence loops between five structures are with the harmonized color.

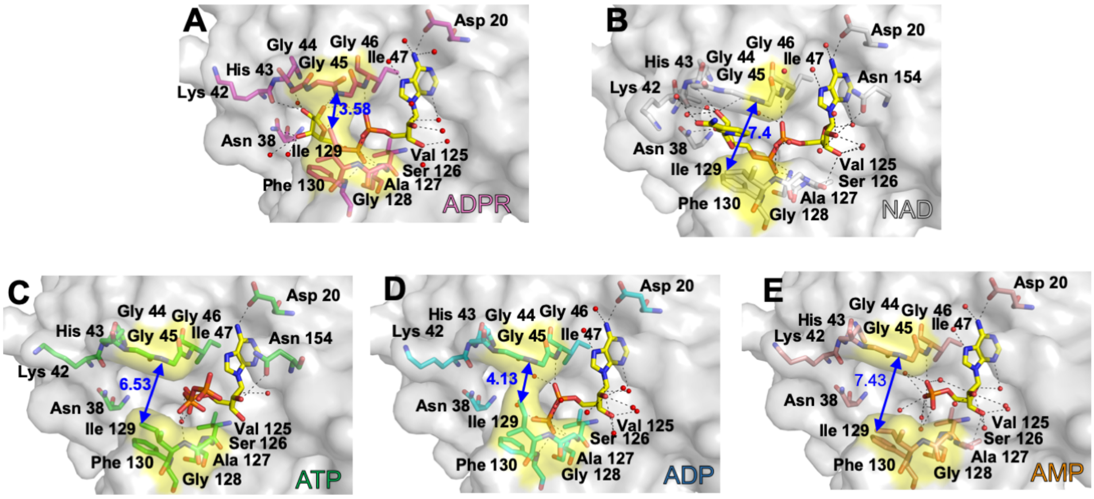

Supplementary Figure S4. Interaction of MERS-CoV macro domain with NAD metabolites. Liquorice-surface representation of the MERS-CoV macro domain binding pocket in presence of (A) ADPR, (B) NAD, (C) ATP, (D) ADP or (E) AMP. Residues important for the interaction are highlighted.

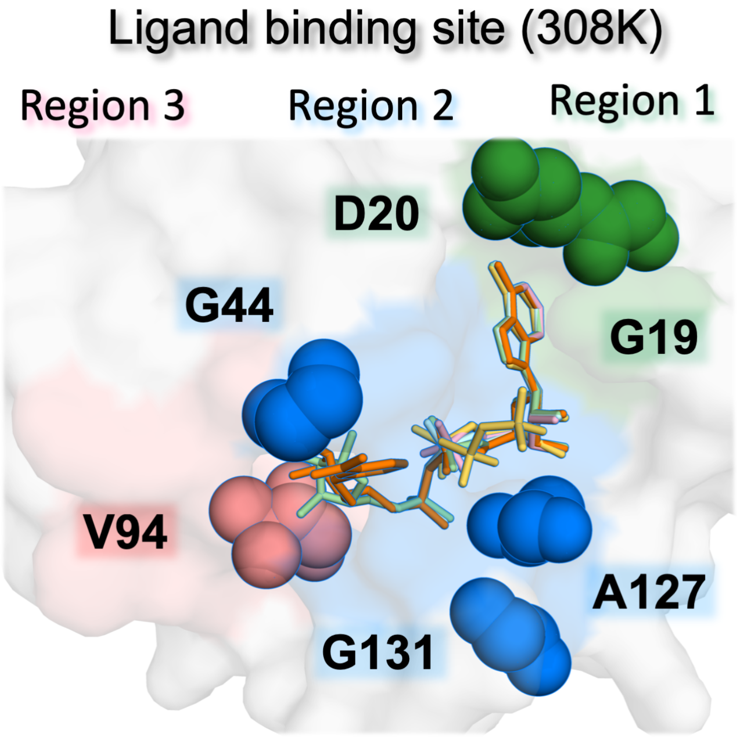

Supplementary Figure S5. The ligand binding site of MERS-CoV macro domain constructed by three regions. Region 1, 2, and 3, who respectively connecting the adenine group, diphosphate group, and distal ribose of ligand, are colored by dark green, marine blue and salmon. NAD metabolites, ADPR, NAD, ATP, ADP and AMP, berried in the ligand binding site are respectively colored by light green, orange, yellow, cyan and light pink. Six amino acids surrounding the ligand binding site are selected to be analyzed in NMR perturbation assays at 308K: G19 and D20, located in region 1; G44, A137 and G131, located in region 2; V94 in region 3.

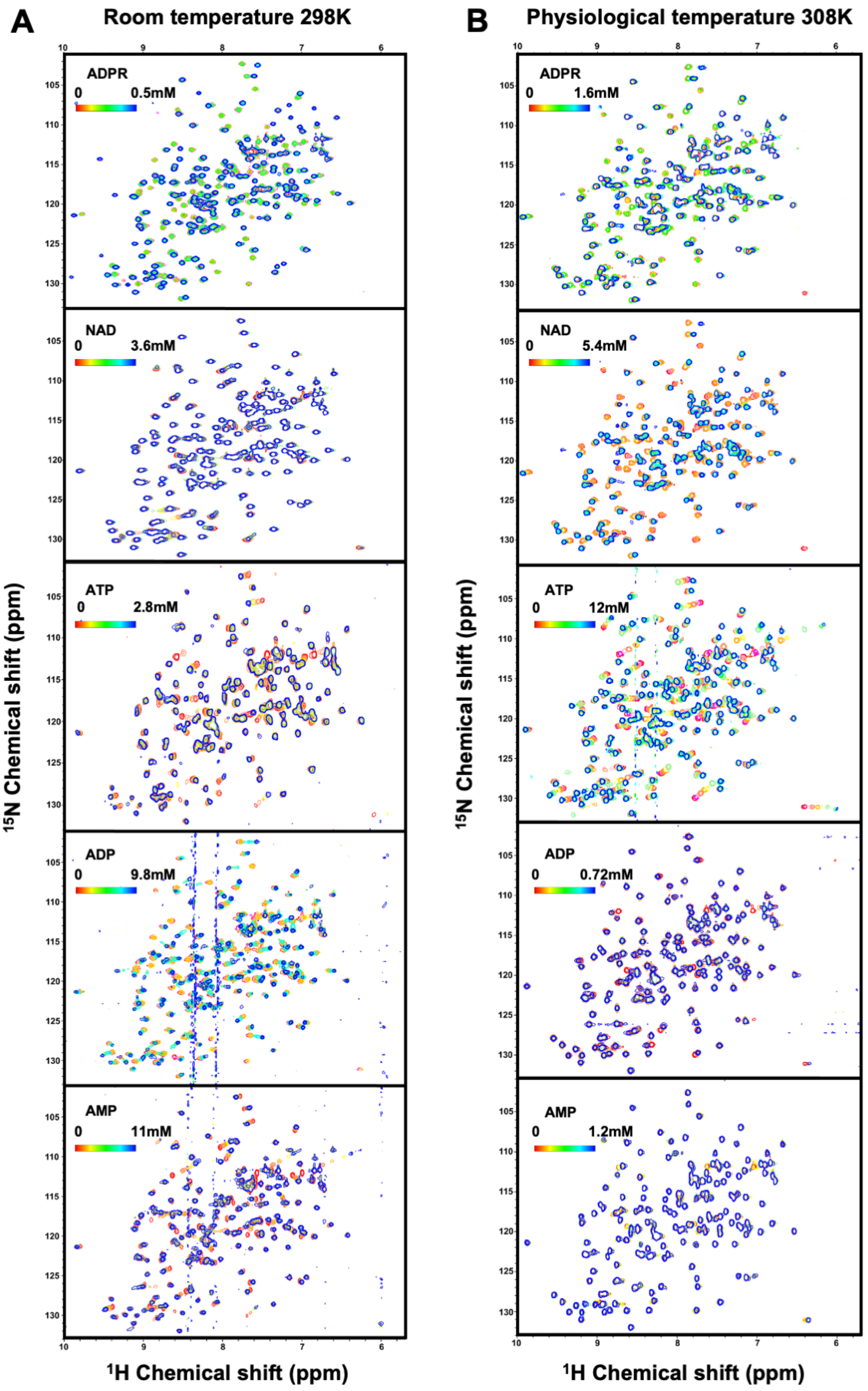

Supplementary Figure S6. NMR perturbation of MERS-CoV macro domain titrated with a series of NAD metabolites at 298K (A) and 308K (B). The shown spectra are overlays of the ^{1}H-^{15}N HSQC spectra of MERS-CoV macro domain alone and a series of NAD metabolites titrations including ADPR, NAD, ATP, ADP, and AMP, respectively.

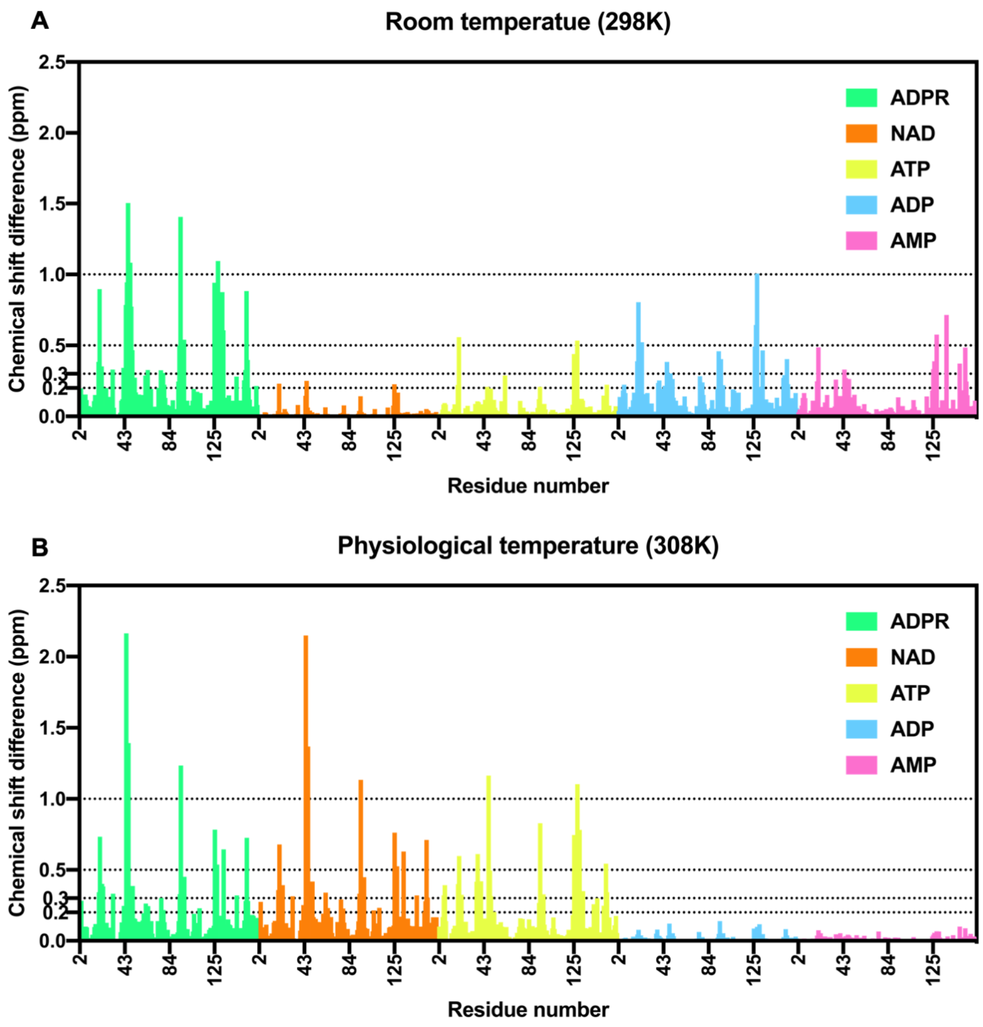

Supplementary Figure S7. Chemical shift difference per residue plot at 298K (A) and 308K (B). Chemical shift difference of protein structures complex with ADPR, NAD, ATP, ADP and AMP are colored in light green, orange, yellow, cyan and light pink respectively.

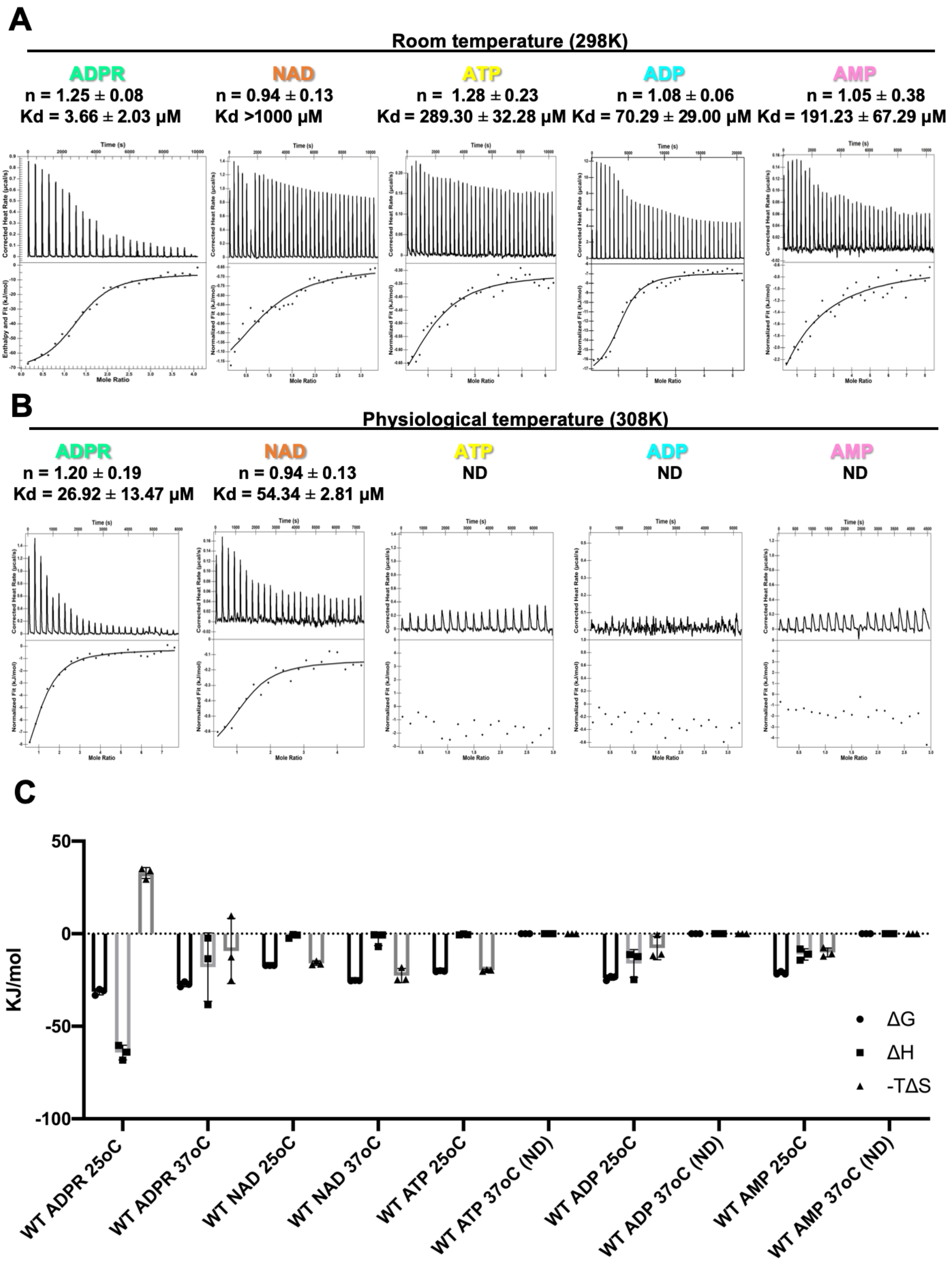

Supplementary Figure S8. ITC analysis of MERS-CoV macro domain with different NAD analogues at 298K (A) and 308K (B). Integration of raw data yielding the heat per mole versus molar ratio. (C) The thermodynamic profiles (free energy (ΔG), enthalpy (ΔH) and entropy (-TΔS), which determined by ITC analysis) of each experiment. Independent experiments n=3.

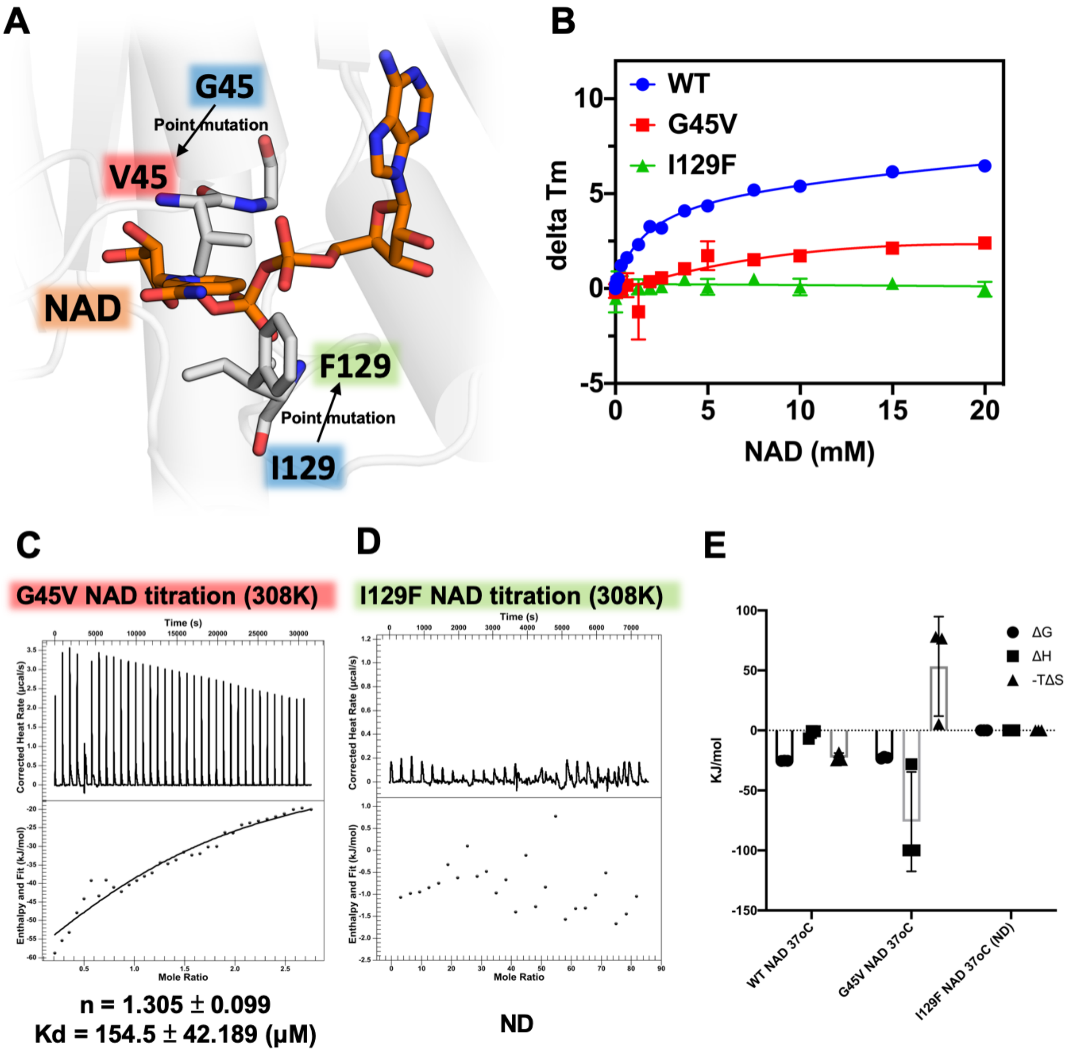

Supplementary Figure S9. Point mutations at distinguished loops 1 and 2. (A) Scheme of the positions of introduced point mutations, G45V and I129F. (B) NAD binding to MERS-CoV macro domain WT and mutants, as probed via DSF. DSF thermal shift assay of 0.2 mM protein incubating with increasing concentrations of NAD. Data are the mean ± SE of the melting temperature difference (delta Tm) from 3 independent experiments. Data were fitting by the means. (C), (D) ITC analyses of NAD titrating to G45V and I129F. Integration of raw data yielding the heat per mole versus molar ratio. (E) The ITC thermodynamic profiles (free energy (ΔG), enthalpy (ΔH) and entropy (-TΔS), which determined by NAD titrations) of each experiment. Independent experiments n=3.

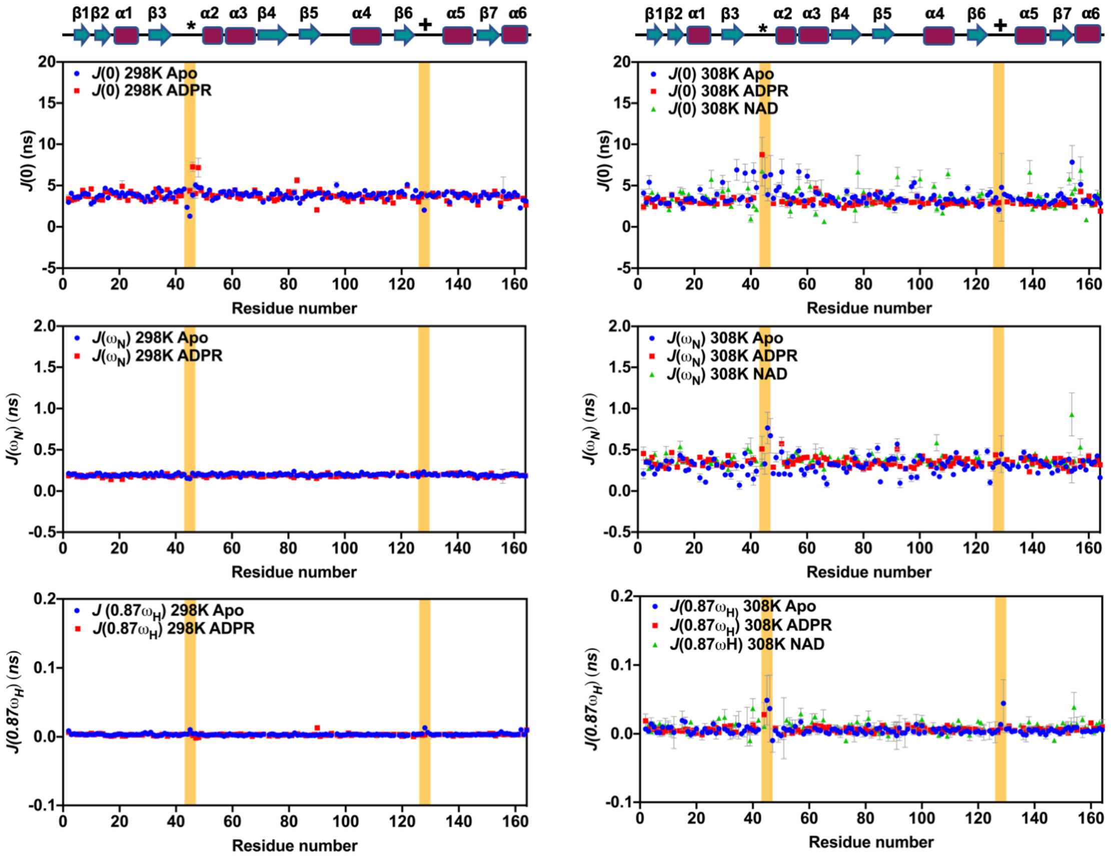

Supplementary Figure S10. Reduced spectral density values of the MERS-CoV macro domain. Left panel presents the $J(0)$, $J(\omega_N)$, and $J(0.87\omega_H)$ values of the apo form and ADPR-bound form of the MERS-CoV macro domain at 298K. Right panel presents the $J(0)$, $J(\omega_N)$, and $J(0.87\omega_H)$ values of the apo, ADPR-bound, and NAD-bound form of the MERS-CoV macro domain at 308K. Secondary structures of the MERS-CoV macro domain are presented at the top of the alignment. Distinguished loops 1 and 2 described in this study are labeled as * and +, and further colored in yellow.

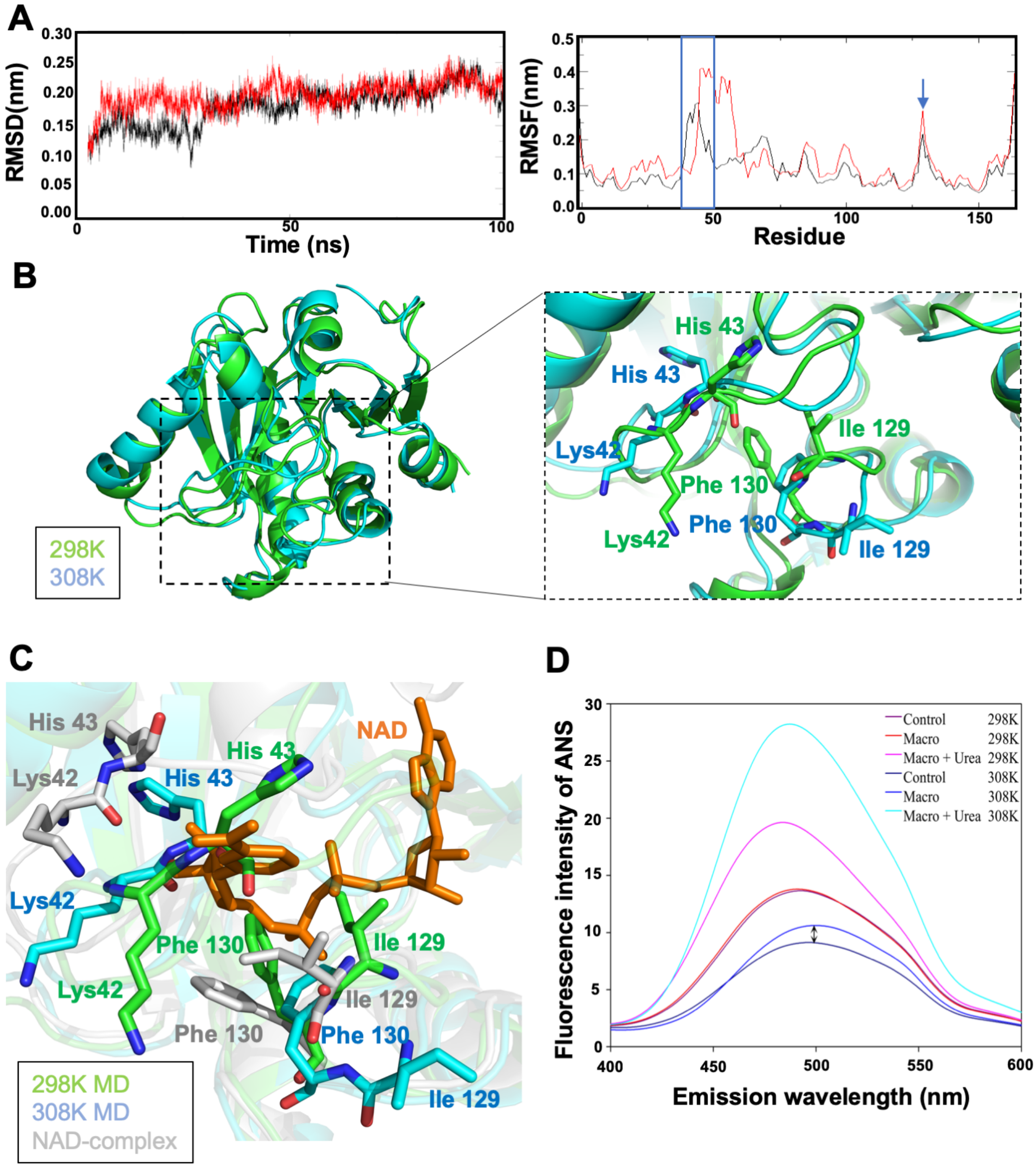

Supplementary Figure S11. Temperature tune of Apo form MERS-CoV macro domain. (A) RMSD and RMSF plots of MERS-CoV macro domain apo form at 298K (black lines) and 308K (red lines). Specific differences of RMSF at two distinguished loops are labeled by a blue box and a blue arrow. (B) Detail difference of side chains of residues at loop β3-α2 and β6-α5 are shown. (C) The temperature induced side chain flipping of residues at loop β3-α2 and β6-α5 in apo form controls the enter of NAD. (D) ANS fluorescence spectra of MERS-CoV macro domain apo form at 298K and at 308K.

Supplementary Information - Uncropped Blots and Gels

Figure 6A α-biotin

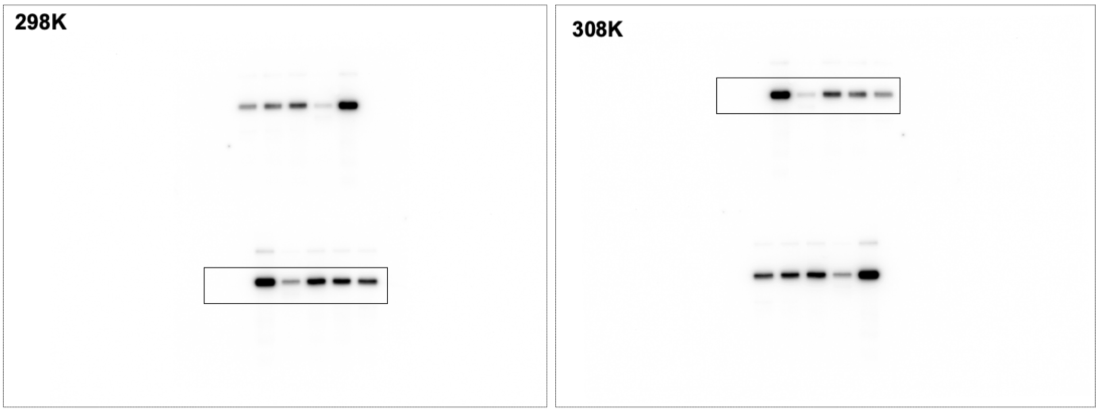

Figure 6A CBB

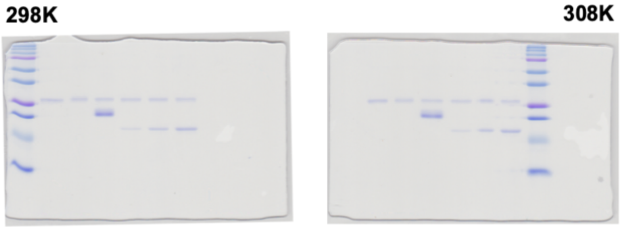

Figure 6C α-biotin

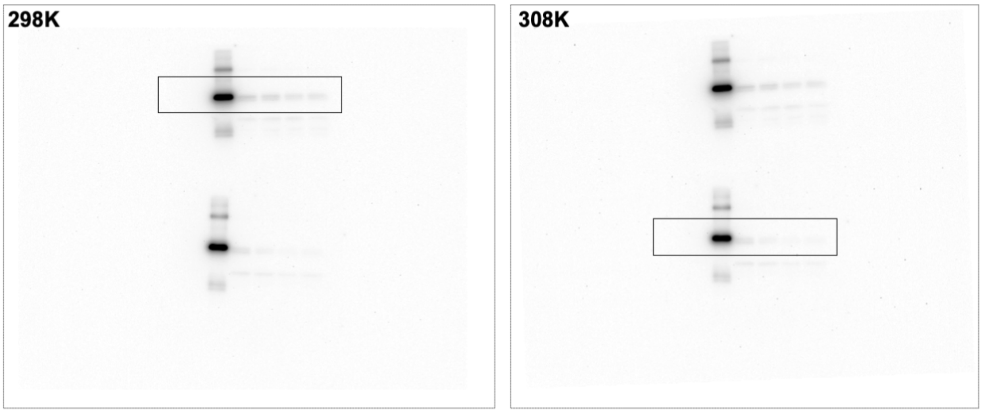

Figure 6D α-biotin

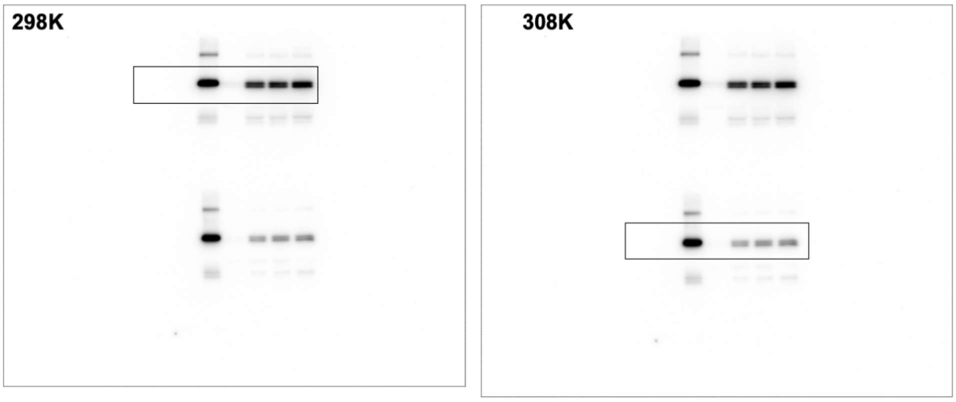

Supplement: Supplementary file 2 — Supplementary Information [file 42003_2020_1633_MOESM2_ESM.pdf]
