# Supplementary material for: Elucidating the tunability of binding behavior for the MERS-CoV macro domain with NAD metabolites
Source: Commun Biol. 2021 Jan 27;4:123. doi: 10.1038/s42003-020-01633-6 (PMC7840908; doi:10.1038/s42003-020-01633-6)
Supplement: Supplementary file 3 — Description of Additional Supplementary Files [file 42003_2020_1633_MOESM3_ESM.pdf]

Description of Additional Supplementary Files

File name: Supplementary Data 1

Description: Source data for charts in the main figures.
